# Simple and rationale-providing SMS reminders to promote accelerometer use: a within-trial randomised trial comparing persuasive messages

**DOI:** 10.1186/s12889-018-6121-2

**Published:** 2018-12-07

**Authors:** Matti T. J. Heino, Keegan Knittle, Ari Haukkala, Tommi Vasankari, Nelli Hankonen

**Affiliations:** 1 Faculty of Social Sciences, University of Helsinki, Helsinki, Finland; 2 Faculty of Social Sciences, University of Tampere, Tampere, Finland; 3 UKK Institute for Health Promotion Research, Tampere, Finland

**Keywords:** Accelerometry, Intervention, Text messaging, SMS, Persuasion, Adherence, Behaviour change, Adolescents, School-based research, Partially randomised trial

## Abstract

**Background:**

Literature on persuasion suggests compliance increases when requests are accompanied with a reason (i.e. the “because-heuristic”). The reliability of outcomes in physical activity research is dependent on sufficient accelerometer wear-time. This study tested whether SMS reminders—especially those that provided a rationale—are associated with increased accelerometer wear-time.

**Methods:**

We conducted a within-trial partially randomised controlled trial during baseline data collection in a school-based physical activity intervention trial. Of 375 participants (mean age = 18.1), 280 (75%) opted to receive daily SMS reminders to wear their accelerometers. These 280 participants were then randomised to receive either succinct reminders or reminders including a rationale. Data was analyzed across groups using both frequentist and Bayesian methods.

**Results:**

No differences in total accelerometer wear minutes were detected between the succinct reminder group (Mdn = 4909, IQR = 3429–5857) and the rationale group (Mdn = 4808, IQR = 3571–5743); W = 8860, *p* = 0.65, CI95 = − 280.90–447.20. Similarly, we found no differences in wear time between participants receiving SMS reminders (Mdn = 4859, IQR = 3527–5808) and those not receiving them (Mdn = 5067, IQR = 3201–5885); W = 10,642.5, *p* = 0.77, CI95 = − 424.20–305.30. Bayesian ANOVA favored a model of equal weartime means, over one of unequal means, by a Bayes Factor of 12.05. Accumulated days of valid accelerometer wear data did not differ either. Equivalence testing indicated rejection of effects more extreme than a Cohen’s d (standardised mean difference) of ±~0.3.

**Conclusions:**

This study casts doubt on the effectiveness of using the because-heuristic via SMS messaging, to promote accelerometer wear time among youth. The because-heuristic might be limited to face-to-face communication and situations where no intention for or commitment to the behavior has yet been made. Other explanations for null effects include non-reading of messages, and reminder messages undermining the self-reminding strategies which would occur naturally in the absence of reminders.

**Trial registration:**

DRKS DRKS00007721. Registered 14.04.2015. Retrospectively registered.

**Electronic supplementary material:**

The online version of this article (10.1186/s12889-018-6121-2) contains supplementary material, which is available to authorized users.

## Background

### Compliance with accelerometer wear instructions

Reliable and valid assessment is necessary when evaluating whether public health policies or interventions change physical activity (PA) levels in the target group. Little consensus exists about what to measure, when, with what and for how long in PA research [1, 2]. While an inability of individuals to accurately remember their past PA and social desirability are clear problems with self-reported PA measures [3], objective measurements of PA (e.g. pedometers and accelerometers) have issues too. Zhuang et al. [4] found that missing accelerometry data was more common in 15- to 17-year-olds than among younger participants, especially during weekends (Sundays in particular), with missing data occurring increasingly from the first recording day to the last. This exemplifies a key issue in measurement: the proportion of an individual’s day or week captured by the measure. An extreme example would be an individual, who only wears the measurement device when undertaking PA. Thus, some guidelines suggest that a person should wear an accelerometer for a minimum of 10 h daily for at least 4 days in a 7-day measurement period in order to obtain an accurate reading of PA [1, 2]. Participants’ compliance with instructions on wearing the accelerometer is clearly very important in obtaining accurate PA measurements [5].

Research on enhancing accelerometer instruction compliance rates is rare [2, 6], particularly among older adolescents. One strategy has been monetary incentives contingent on proper wear-time [7]. Sallis et al. [8] used an alternative strategy, asking participants to re-wear the accelerometer if they had not worn it for at least 5 valid days (> 10 valid hours of data) or a minimum of 66 valid hours across 7 days.

Barak et al. [9] suggest that new opportunities to promote compliance—such as text messaging (SMS; Short Messaging Service)—may be more reliable and effective than traditional methods, such as written or verbal wear instructions by the investigator. Zhuang et al. [4], too, recommend SMS reminders. Toftager et al. [10] used SMS reminders to increase compliance but did not report effects or acceptability. In a self-selected Irish sample of adolescents [11], daily SMS reminders were associated with putting on the accelerometer in the morning, but not in increased overall compliance (defined as valid days of data or minutes of non-wear). The study did not report levels of wear or effects of the reminders. The discrepancy between remembering to put on the device and actually wearing it for a sufficient amount of time indicates that these may be separate behaviors.

### Compliance and the ‘because-heuristic’

Since the classic “Xerox machine study” by Langer, Blank and Chanowitz [12], providing reasons for compliance has been discussed in the social influence literature. The study indicated that placebic or pseudo-reasons [13] (“*Excuse me, I have 5 pages. May I use the xerox machine, because I have to make copies?*”; 93% compliance) could result in similar compliance rates as actual reasons (“[…] *because I’m in a rush?*”; 94% compliance) compared to the request only condition (“*Excuse me, I have 5 pages. May I use the xerox machine*”; 60% compliance). Pratkanis (2007), identified “placebic reasons” in his index of social influence tactics, but called for further research into the subject. Less careful are Cialdini, Goldstein and Martin [14], who tout the “unique motivational influence of the word *because*”, basing their claims on the importance of reasoning in social influence. To this day, the xerox machine study remains cited in the press as an example of the power of the word ‘because’ [15–18].

A well-known principle of human behavior says that when we ask someone to do us a favor we will be more successful if we provide a reason. People simply like to have reasons for what they do. [19]

Following the terminology used by Key, Edlund, Sagaring and Bizer [20], the phenomenon of increased compliance by providing reasons is referred to as “the because-heuristic.” Let us accordingly define the *naïve because-heuristic* as “reasons increase compliance.”

In the Langer, Blank and Chanowitz study 1, this effect of reasons increasing compliance was only found when the confederate asked for ‘a *small* favor’ (five instead of ten pages, translating to effect sizes of d = 0.87 and d = 0.13, respectively) [12]. Still, the results in general, as well as their implications have been questioned [21, 22]. A study by Folkes suggests, that instead of the size of the request, the effect is moderated by controllability [21]. Pooling Folkes’ reason conditions results to an effect size of d = − 0.026, speaking against the quote above, and pointing out that the “power of reasons” effect is malleable, in the least.

To our knowledge, only one published direct replication of the Langer, Blank and Chanowitz study 1 exists [20]. The main effect of the study replicated (d = 0.67 for placebic over no reason and d = 0.69 for real over no reason conditions), although over 20% (34 out of 163) of the participants needed to be excluded for various reasons. Lack of published replication studies, of course, is not new in the field of psychology [23].

In a conceptual replication of the phenomenon, in small request conditions, reasons (either placebic or real) increased compliance by an equivalent of d = 0.43 (calculated from Table 1 of [24]) when including their additional persuasion group and d = 0.22 when excluding it. Another conceptual replication [25] found d = 0.15 for requests perceived as small, and d = 0.21 for requests perceived as large (as calculated from Figure 3 of [25]).

These studies seem to temper earlier claims for the power of reasons in increasing compliance. In contrast to the *naïve because-heuristic*, let us define the *weak because-heuristic* as “reasons increase compliance, but only if the perceived favour is small”.

This study will investigate the effects of the because heuristic on compliance with the physical activity measurement procedures in the context of baseline measurements of a large school-based intervention.

### The Let’s Move It cluster randomized trial

Inadequate PA predicts increased morbidity and mortality in people of low socioeconomic status (SES) [26], with SES differences in PA emerging already in adolescence [27]. Finnish vocational school students are less physically active than those in high school [28]. The Let’s Move It intervention aimed to increase PA and decrease sedentary behaviors in older adolescents in vocational schools.

The current study was conducted as a sub-study of the cluster randomised effectiveness evaluation trial of the Let’s Move It intervention [29]. In a preceding feasibility study [30], participants’ accelerometer wear times were suboptimal; 47% (18/38) of baseline participants reached the cutoff of 10 h per day for at least 4 days, 63% (17/27) for the first and 75% (9/12) for the second follow-up. A frequently cited explanation for not wearing the accelerometer was forgetting to put on the device.

### Aims and hypotheses

In this within-trial study, we investigate SMS-reminder strategies to improve the duration of accelerometer wear time. The literature cited previously lead us to hypothesise that reminders would increase accelerometer wear time and that citing reasons would amplify the effect. In addition to daily wear hours, we are interested in the number of days our participants provide valid activity data (i.e. days of ≥10 h of activity data). The target behavior is thus twofold: 1) putting on the accelerometer in the morning for as many days as possible, 2) wearing the accelerometer for as long as possible in the waking hours each day. In this study, two main research questions are posited:

1. Are SMS-reminders associated with greater accelerometer wear times?

The current study investigated this by comparing the compliance rates across a) participants who opted to receive SMS reminders to wear their accelerometer, and b) participants who opted not to receive the reminders (non-randomised control group). If forgetting is an important reason for non-compliance, in the absence of intervening factors, reminders should increase compliance.

Statistical hypothesis H_1_: Those who receive SMS reminders will have higher accelerometer wear times than those who do not.

2. Does offering reasons to comply affect accelerometer wear time?

If reasons increase compliance, SMS reminders containing reasons to wear an accelerometer should lead to greater compliance.

Statistical hypothesis H_2_: Those who receive reasons in the SMS reminders have more minutes of accelerometer wear and more days of valid data (≥10 h of activity) than those who do not receive reminders containing a reason.

An additional research question, on whether providing reasons to comply with accelerometer wear increases trial retention, is omitted here. These null results are reported in [31].

## Methods

**Fig. 1:**
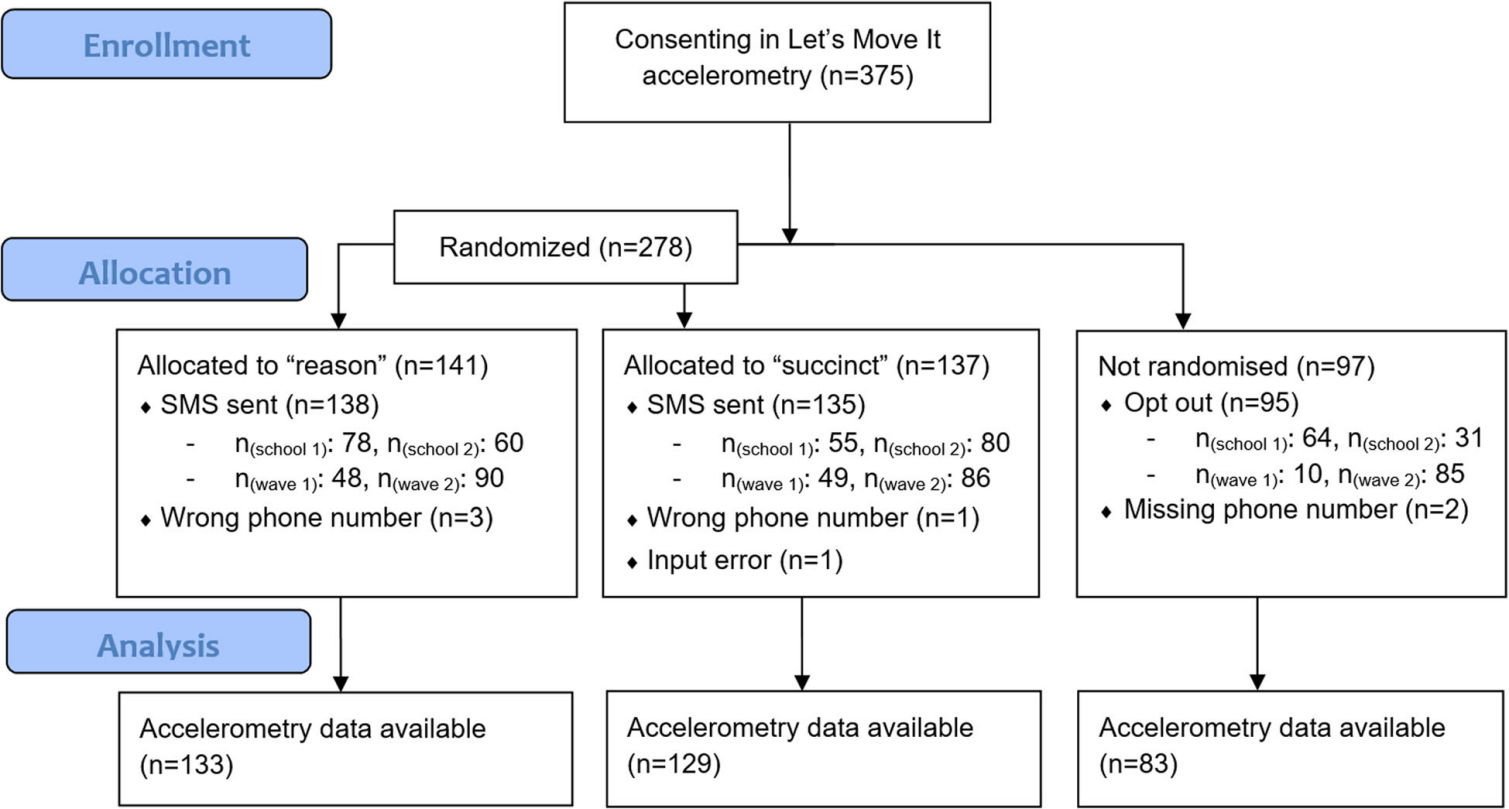
CONSORT flow diagram

The design of this study was a within-trial, outcome-assessor blinded, partially randomised controlled trial (RCT). In addition to the randomised experiment between two message types, quasi-experimental data were acquired from a self-selected opt-out arm (see Fig. 1). This study was conducted during the baseline assessment of the first two recruitment waves (out of six) of the Let’s Move It cluster-randomised controlled trial [29]. This article is based on unpublished work available at https://osf.io/89mhu/. Additional information on methods and results, in addition to all analysis code, can be found in the supplementary website at https://git.io/vNl8X (permalink provided in [32]).

### Participants and sampling procedures

To be included in the study, the participants had to fulfill inclusion criteria of the Let’s Move It study [29] and had to have consented to the accelerometry measurements: all were at least 16 years old and were vocational school students. The reminder arms consisted of the participants who opted in to receive reminders for accelerometer wear.

During baseline recruitment of the first two recruitment waves of the Let’s Move It trial, students in two vocational schools were approached during class and informed about their school’s study participation in the study. After the invitation to participate in the main trial and collection of signed informed consent forms, those who consented were given an online questionnaire to complete. Details of trial procedures are reported in the protocol [29].

After 1–3 days, research assistants gave the participants a waist-worn accelerometer (Hookie AM 20, Traxmeet Ltd., Espoo, Finland) and instructed them on how to wear it for a duration of seven consecutive days (including the day of receiving the device). The used Hookie accelerometer is a tri-axial accelerometer that collects data at 100 Hz sampling rate without preprocessing. The measurement range of the accelerometer is ±16 g and the resolution is 4 mg (milligravity). The Hookie accelerometer employs the same tri-axial acceleration sensor component (ADXL345; Analog Devices, Norwood MA) that is used in widely used research-grade accelerometers [33]. The validation of the Hookie accelerometer has been reported in both children [34] and adults [35] in studies comparing analysis of raw acceleration data from different accelerometers.

When participants received the accelerometers, they were asked whether they would like to receive SMS messages to help them remember to put it on every morning. Those who consented to the messages were subsequently randomised to one of two message conditions, and those who opted not to receive the reminders were treated as a self-selected control arm.

After 7 days, participants returned their devices to research assistants and were asked to fill out a short questionnaire assessing process measures (see Additional file 1: Appendix S1 and Additional file 2: Appendix S2.

### Random assignment

Participants were assigned to the reason and succinct arms after they were recruited. The first author extracted the phone numbers from the list and used R code to create an amount of random numbers equal to the number of new participants. The vector of random numbers was then assigned to the participants. Participants with a number equal to or smaller than the median of the vector were allocated to the reason-condition. Others were allocated to the succinct condition. Research assistants working in the field to assess were blind to group allocation. Recruitment and randomisation took place on the same day, and restrictions such as blocking or stratification were not used.

Recruitment took place in two waves, alongside the recruitment of the main trial. In order to increase the rates of participants opting in for the reminders, the recruitment prompt was slightly modified for the second wave. The research assistants presented the SMS reminders as the default option, and asked whether this is acceptable to the participants.

Random assignment was not visible to the participants and the research assistants did not mention that different kinds of messages were going to be sent. The statistician who analysed the raw accelerometer data was blind to group assignment.

### Interventions

An important issue regarding the current study was to avoid tampering with the effects of the main trial. In other words, it should not affect main trial outcome measures in any other ways except for increased data quality. Care was taken to formulate the SMS messages to not pressure participants or provoke changes in main trial outcome measures such as PA.

We altered a previous procedure [11] by varying the message content slightly each day to reduce habituation and thus expected to increase the chances of the message being read, for both arms.

The two arms received different message content.

- **Succinct reminder condition:** 1. a greeting – 2. a reminder – 3. a thank you
- **Reminder and reason:** 1. a greeting – 2. a reason beginning with “Because…”, followed up with a reminder – 3. a thank you

Messages are presented in detail in Table 1 below.

**Table 1:** SMS content, translated to English

Morning	Reminder with rationale (the “because heuristic”)	Succinct reminder
1st	Morning! Because your participation is precious, please remember to put on the motion measurement device and wear it until you go to sleep (except in the shower etc.) - thanks!	Morning! This is a reminder to put on the motion measurement device and wear it until you go to sleep (except in the shower etc.) - thanks!
2nd	Hi! Because you’re aboard in producing very important knowledge, please remember to put on the motion measurement device now and wear it as instructed until you go to sleep. Thanks a lot!	Hi! Please remember to put on the motion measurement device now and wear it as instructed until you go to sleep. Thanks a lot!
3rd	Hello! Because the study wouldn’t succeed without your help, please remember to put on the motion measurement device again and wear it until you go to sleep (except in the shower etc.) - thanks!	Hello! Please remember to put on the motion measurement device again and wear it until you go to sleep (except in the shower etc.) - thanks!
4th	Morning! Because the data you gather is highly valued, please remember to put on the motion measurement device and wear it until you go to sleep. Thanks (we’re already past midpoint)!	Morning! Please remember to put on the motion measurement device and wear it until you go to sleep. Thanks (we’re already past midpoint)!
5th	Howdy! Because your participation produces very important knowledge, please remember to put on the motion measurement device and wear it until you go to sleep (except in the shower etc.) - thanks!	Howdy! Please remember to put on the motion measurement device and wear it until you go to sleep (except in the shower etc.) - thanks!
6th	Hi! Because even this last day is important, please remember to put on the motion measurement device and wear it until you go to sleep. Return the motion measurement device to school tomorrow - thanks!	Hi! Please remember, even on this last day, to put on the motion measurement device and wear it until you go to sleep. Return the motion measurement device to school tomorrow - thanks!

We sent the messages using an SMS Gateway device MT-SF100-G-EU (MultiModem iSMS Server 1-port) by Multi-Tech Systems (http://www.multitech.com/brands/multimodem-isms). We used a manufacturer-designed guided user interface for the first recruitment wave and a custom interface designed by a local service provider for the second wave.

### Registration and deviations from registered plan

The study plan was reviewed by the Ethics Committee for Gynaecology and Obstetrics, Pediatrics and Psychiatry of the Hospital District of Helsinki and Uusimaa (decision number 367/13/03/03/2014).

Official public registration in the German Clinical Trials Register (DRKS-ID: DRKS00007721) was completed 3 months after recruitment of the first wave had been initiated, but before data was available. Pre-registration (before starting data collection) failed due to lack of available resources at the time.

The original plan was to establish the additive effect of messages containing a reason and those not containing one over a no-message condition during the baseline measurement of the first batch. With the sample size we expected (*n* = 140), we would have had over 95% power to detect an effect of d = 0.6 (slightly smaller than the one discovered in the Langer, Blank and Chanowitz replication [20]). We had then planned to pit the more successful message type against a third message in the second wave. Instead of going forward with the plan of using a third message, we made the decision to gather another wave of participants with the same message types after the data from the first wave was analysed. This was due to the fact that, contrary to our expectations, no difference between the two messages was detected. This is important to note, as it means we can no longer rely on a long-term error rate of 5% [36] and—as *p*-values depend on the sampling distribution—default *p*-values from common statistical programs no longer apply [37].

To address the issue of inadequate reporting in the sciences [38], the current report complies with the Consolidated Standards of Reporting Trials (CONSORT) statement [39]. Contributor roles are clarified in Additional file 3: Appendix S3, according to a taxonomy for this purpose [40].

### Outcomes

#### Primary outcome measures

Primary outcome measures were 1) accelerometer wear time minutes and 2) days with ≥10 h of valid accelerometer data. As this trial was conducted within a larger trial, several other measures were collected and are listed in the Let’s Move It protocol [29]. The main trial used a 3-axis accelerometer with a 2GB internal memory (Hookie Meter v2.0, Hookie Technologies Ltd., Espoo, Finland). The activity data was registered using raw data and a 100 Hz sampling rate.

#### Implementation assessment measures

A one-page questionnaire (Additional file 1: Appendix S1; translation in Additional file 2: Appendix S2) was used to gain additional insight into the reception of the messages.

**Self-reported message receipt.** As we could not gather objective log data on the number of messages opened, we asked participants to assess on how many mornings they had opened and read the SMS. Response options were: Not on a single morning, On 1 morning, On 2–3 mornings, On 4–5 mornings and Every morning.

**Manipulation and contamination check.** As participants were randomised individually, as opposed to clusters at school class level, discussing the SMS messages with their classmates could have led to students finding out that not everyone received the same messages, and perhaps also reveal the study hypotheses. We attempted to gauge the extent of this by asking them how often they had discussed the messages with peers. Response options were: Not once, Once, 2–3 times, 4–5 times and More often.

**Acceptability of SMS message content** was assessed by asking the participants, how much they agree with the statement “I was satisfied with the content of the messages”. Response options again had a 5-point scale: Completely disagree, Somewhat disagree, Do not agree nor disagree, Somewhat agree and Completely agree.

### Statistical analyses

All non-Bayesian analyses were conducted using RStudio running R [41, 42]. Plots were drawn using R packages ‘ggplot2’ [43] and ‘yarrr’ [44]. Distributions between the reason and succinct groups in the implementation assessment questions were compared using the chi-square test.

Accelometer wear times were analysed using bootstrapping methods. A 95% bootstrap confidence interval for a mean can be acquired by resampling observed data to simulate a sampling distribution, obtaining the values for the 0.025th and 0.975th percentiles of resampled means [45]. A kernel density plot, bootstrap confidence interval and a bootstrap test of equivalence were conducted using R package ‘sm’ [46] for differences of distributions of the two reminder arms. Wilcoxon rank sum test with continuity correction was used to compare medians between groups.

ANOVA for equivalence of means between the two reminder groups and the no-reminder group, as well as its illustration, was performed using R package ‘userfriendlyscience’ [47]. Additionally, a MANOVA with wear time minutes and wear days with valid data as dependent variables, and SMS group as an independent variable, was used to test robustness of results.

A 95% Bayesian Highest Density Interval (HDI) [44] of the means of valid wear days was plotted using R package ‘yarrr’. HDI refers to the most likely population parameter values (here: means) given the data; information which is not delivered by frequentist confidence intervals [48, 49].

#### Bayes factors

Due to our sampling methods (e.g. decision to collect more data was based on observed data), traditional frequentist statistics faced limitations. Thus, we also calculated Bayes Factors [50–52] for our main outcome measures. A Bayes factor BF_01_ is essentially the ratio of two likelihoods, answering questions such as “Given the data, how many times more likely is the null hypothesis, compared to a specific alternative hypothesis”. We used the R package BayesFactor [53]; For comparing means, this package assigns the alternative hypothesis a Cauchy prior. We used a prior scale of 0.3, in accordance with common effects in health psychological research [54]. This reflects a prior belief that 50% of the effects lie between d = − 0.3 and 0.3. For contingency tables, priors are described in Jamil et al. [55]. The minimum value is 1, and an increase reflects the belief, that the distribution of observations in the given categories under H1 is relatively more similar to H0. Additional information on inference using Bayes Factors, and prior robustness checks are found in the supplementary website (https://git.io/vNl8X).

#### Equivalence testing

In the frequentist statistical paradigm, support for the null hypothesis is indicated by the practice of equivalence testing [56]. For a difference between means, one essentially first establishes a region of equivalence to zero, then conducts and combines two t-tests. The first one tests whether the effect is higher than the lower bound (in our case, − 0.3), and the other tests whether the effect is smaller than the higher bound (in our case, 0.3). The tests were conducted using R package “TOSTER” [57].

We did not conduct multi-level analyses to account for the intra-class correlation of 0.09 for total accelerometer wear time. Heterogeneity analysis is presented in the supplementary website (https://git.io/vNl8X) file under “Heterogeneity among clusters”.

Using standard deviations estimated from feasibility study [30] data, we determined a practically significant effect size for wear time hours to be d = 0.42 – enough to bring a person from 9.5 h of daily data to reach the cutoff of 10 h. For our purposes, we decided to consider effect sizes between − 0.3 and 0.3 as equivalent to zero. Additional details are presented in the supplementary website under “Statistical power”.

**Fig. 2:**
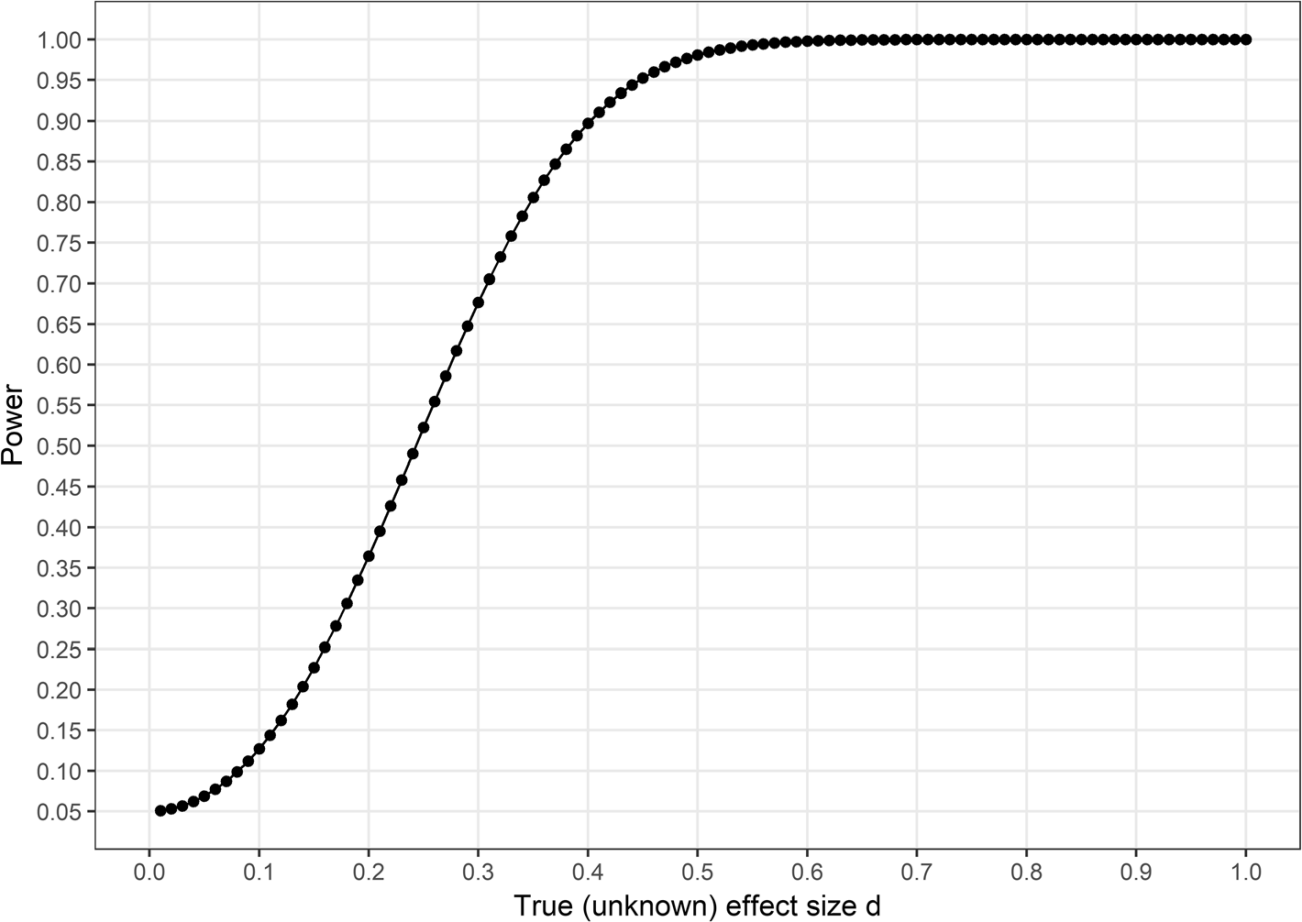
Statistical power, t-test for an unknown real effect

Analysis regarding statistical power is presented in Fig. 2, holding alpha constant at 0.05 and sample size at achieved levels. As seen from the figure, we had 90% power to discover an effect of size d = 0.39, 80% to detect d = 0.3, 60% to detect d = 0.27 and 40% to discover an effect of d = 0.21. Thus, type 2 error probabilities were small for effects near our defined minimal effect size of interest, but high for small effects.

We also evaluated Type S and type M error probabilities [58], and the v-statistic [59]. The analysis is presented in the supplementary website (https://git.io/vNl8X). In brief; our design was relatively well-equipped to handle medium-sized effects, but is subject to considerable bias under small effects.

## Results

### Descriptive data

A participant flow diagram presented in Fig. 1 indicates how the messages were sent to almost all participants as intended.

Of the 375 participants consenting to accelerometer measurements as part of the main trial, 95 opted out of receiving reminders and an additional 7 did not receive messages due to technical difficulties. In the end, the SMS messages with reasons were sent to 138 and the succinct messages to 135 participants. Consent rate for reminders was 54% (101 out of 186) in the first wave and 95% for the second wave (179 out of 189).

Table 2 shows the sample characteristics for the baseline data.

**Table 2:** Sample characteristics

	SMS group				
	Reason	Succinct	Opt out	Send failed	Total
Total n	138	135	95	7	375
Weartime data available	133	129	83	7	352
Female	28%	30%	27%	43%	30%
M age (SD)	17.9 (1.8)	18.2 (2.6)	18.9 (4.3)	18 (1.4)	18.3 (2.9)

Note: One person from both SMS groups missed the first message due to phone number imputation failure. This was considered to be of no practical consequence and they were counted as having received their intervention as planned

### Implementation and process measures

**Fig. 3:**
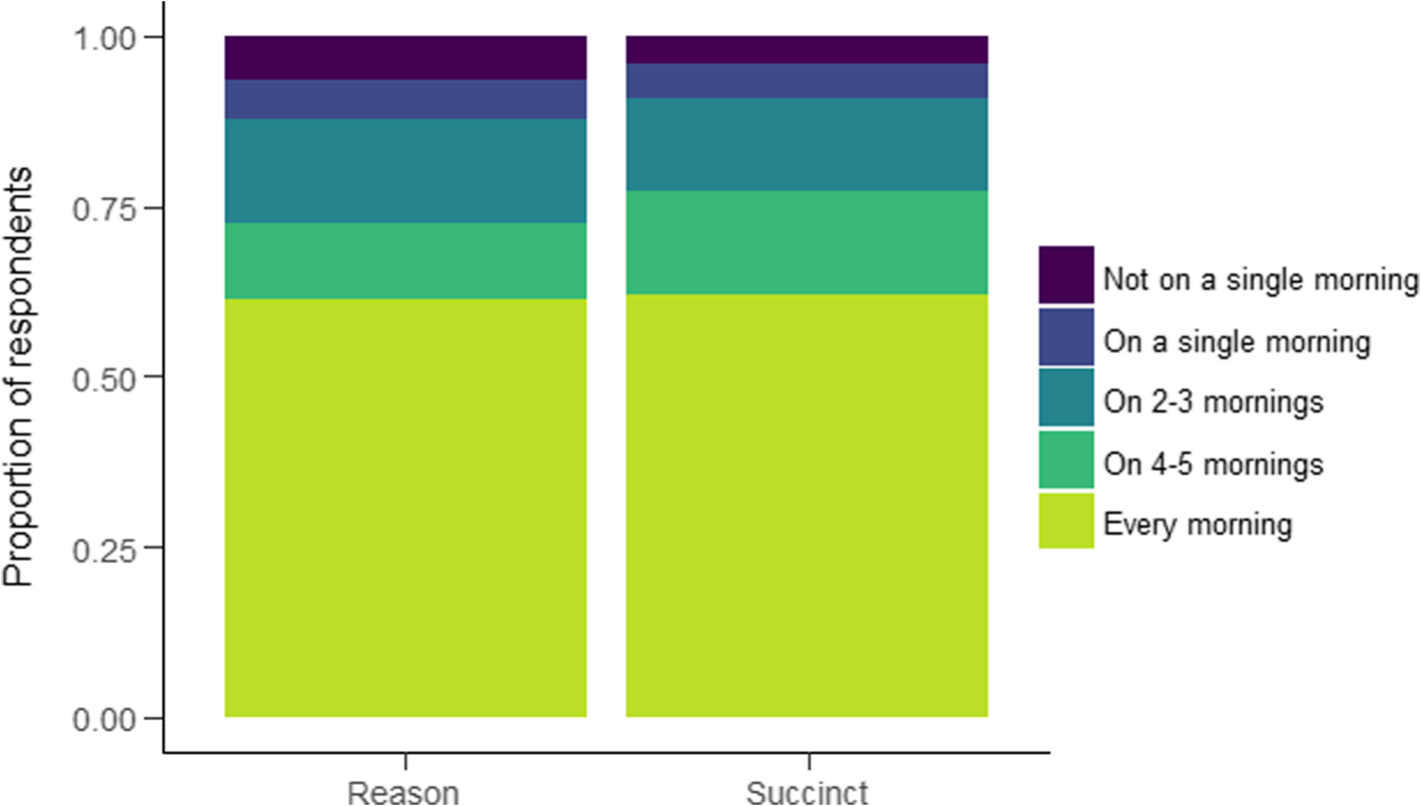
Opening and reading the SMS. Item stem: “I opened the SMS and read it on the morning it was sent.”

**Fig. 4:**
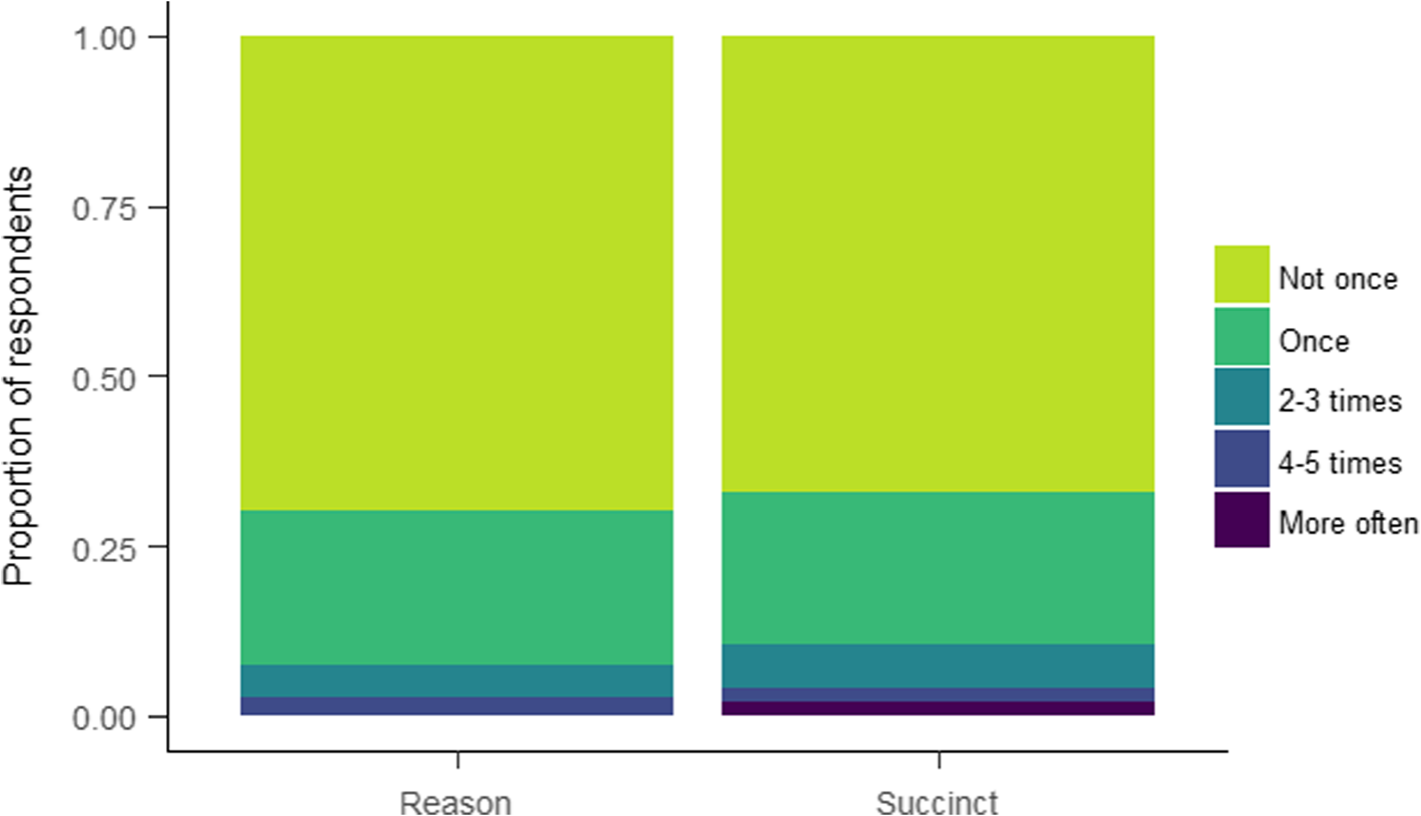
Discussing the SMS with peers. Item stem: “I discussed the content of the messages with my peers at school.”

Manipulation and contamination check, as well as satisfaction with the messages and discussing their content are presented in the supplement. In brief, we did not detect differences across any groups, with Bayes Factors indicating strong support for the null hypotheses. As shown in Fig. 3*.* Seventy four point nine percent of respondents reported having opened and read the SMS at least four mornings. Discussing the content of the messages with peers was not common; 91.1% answered having done so never or just once Fig. 4.

Open comments did not reveal unforeseen negative effects. In addition, 13% (9 out of 70) of participants who answered the question explicitly added, that remembering to wear the device was due to receiving the messages.

### Wear times

#### Wear time minutes

**Fig. 5:**
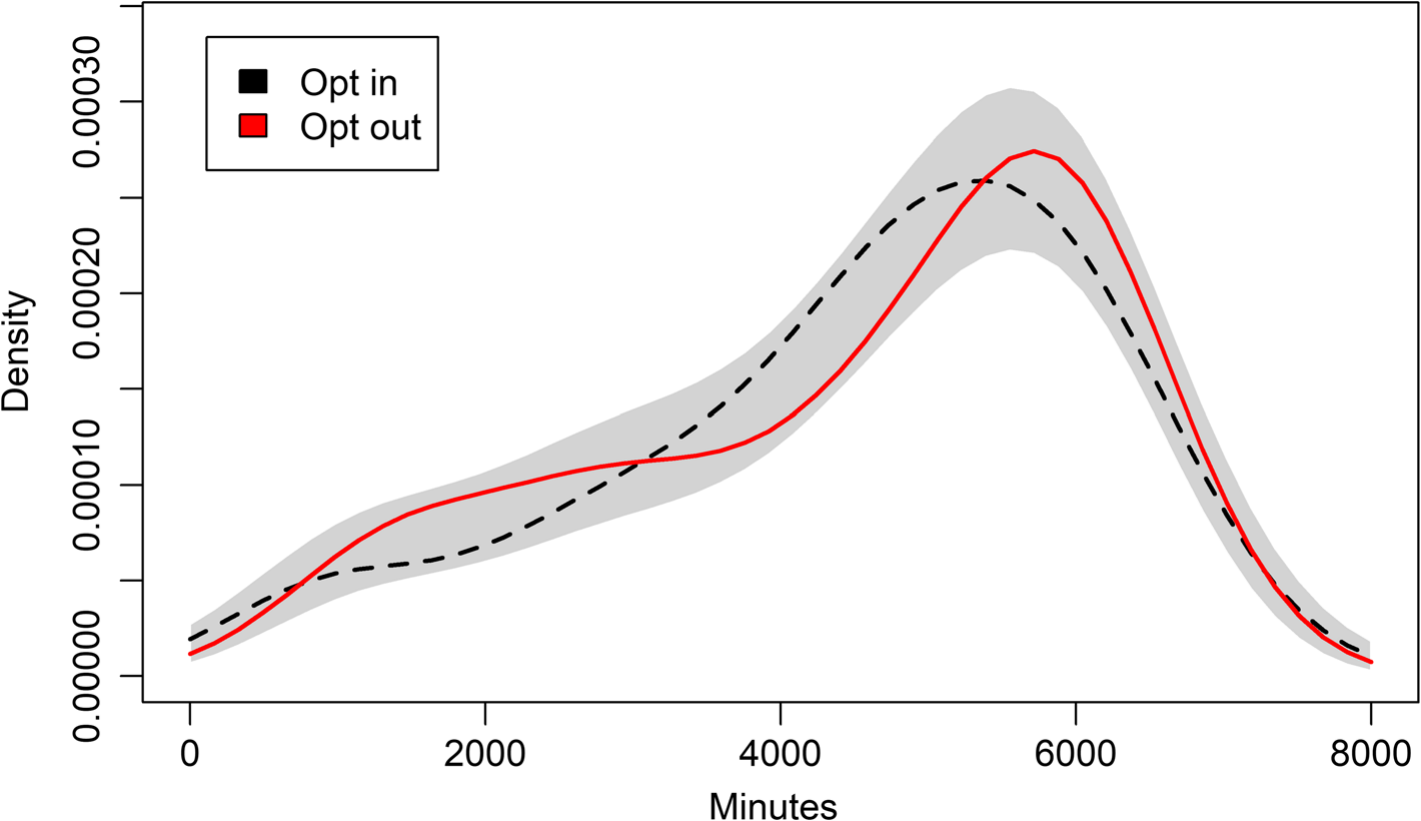
Total wear time in minutes (dashed line for the reason condition, solid for succinct). Grey band around the kernel density plots refers to 95% likelihood of containing the true density plot, if the two lines were generated by data from the same distribution. Mean (SD) Reason: 4549.57 min (1642.14), *n* = 133. Mean (SD) Succinct: 4479.65 (1616.04), *n* = 129

Accelerometer wear times did not indicate meaningful differences between groups (see Fig 5)

Bootstrap tests of equal densities indicated no differences in total wear time minutes between the two message types (*p* = 0.28), nor between those who received and did not receive messages (*p* = 0.35). Wilcoxon rank sum test showed no differences in distributions between message groups (W = 8860, *p* = 0.647, CI95 = − 280.90–447.20) or whether one opted in the messages or not (W = 10,642.5, *p* = 0.771, CI95 = − 424.20–305.30). Differences were neither detected between the two schools (W = 17,398.5, *p* = 0.051, CI95 = − 1.60–619.60) or recruitment waves (W = 17,310.5, *p* = 0.067, CI95 = − 19.0–586.3).

**Fig. 6:**
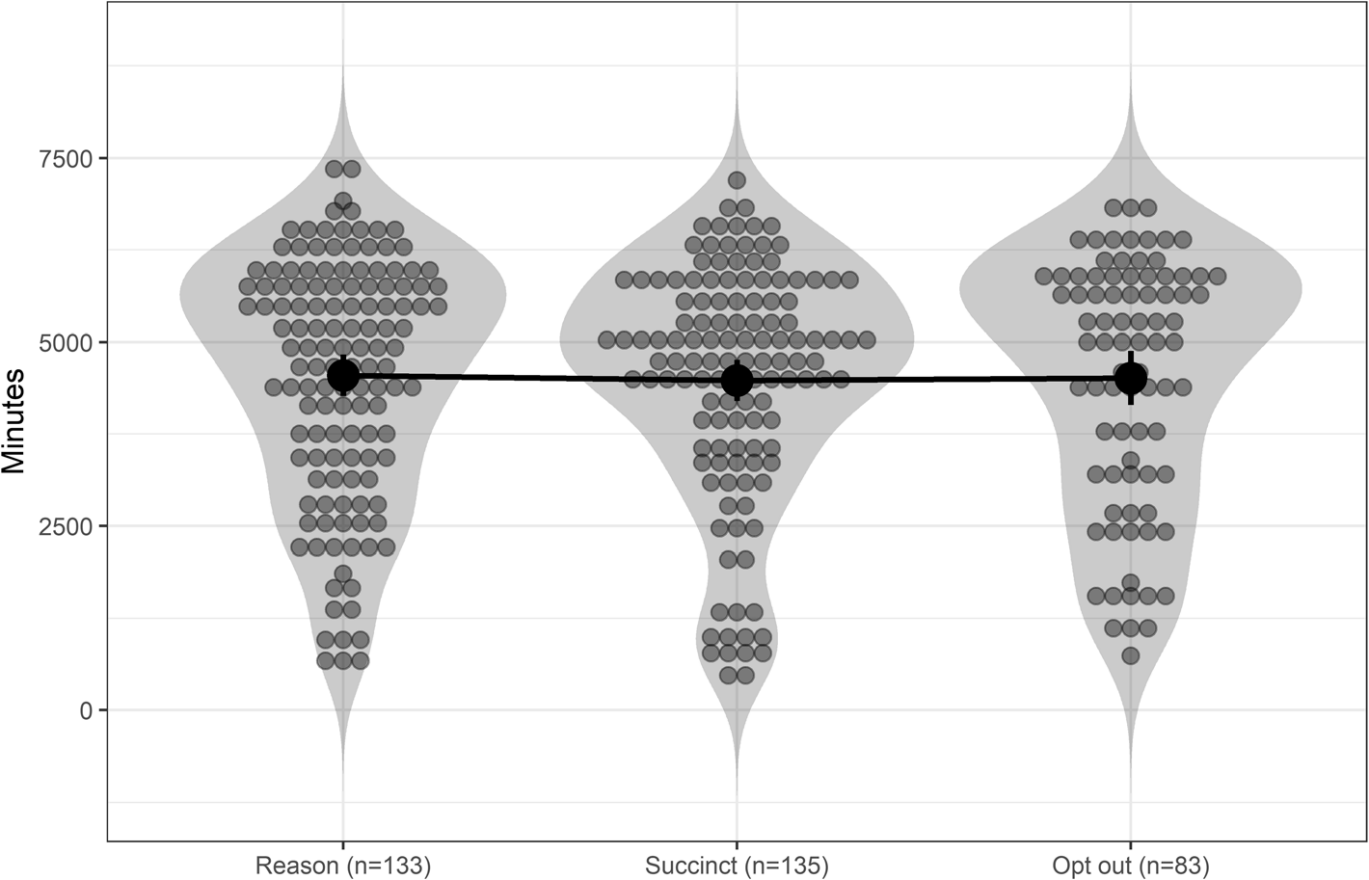
Means and the total wear time distributions of the three groups. Error bars indicate 95% confidence intervals. No differences are detected

The violin plots in Fig. 6 illustrate how wear times in all three groups are distributed. Bayesian ANOVA gives us BF_01_ = 12.05, indicating strong evidence for equivalent means, against a model where all means are unequal. Prior robustness graph (see supplement) starting from *r* = 0 depicted a convex function, where BF_01_ rises to 10 at *r* = 0.27 and reaches 422.34 at *r* = 2.00. Furthermore, BF_01_ relative to an ordered model of Reason > Succinct > Opt out was 23.07 (see section “Interpreting Bayes Factors” in the supplementary website (https://git.io/vNl8X).

Equivalence tests indicated, that the mean wear time differences between message types (69.92 min, 90% CI [− 262.37; 402.21]) and the reminder/opt out groups (1.98 min, 90% CI [− 347.12; 351.08]) were statistically significantly larger than d = − 0.3 and smaller than d = 0.3. In other words, the effect size for the difference in means was deemed less than |0.3|.

#### Valid measurement days

**Fig. 7:**
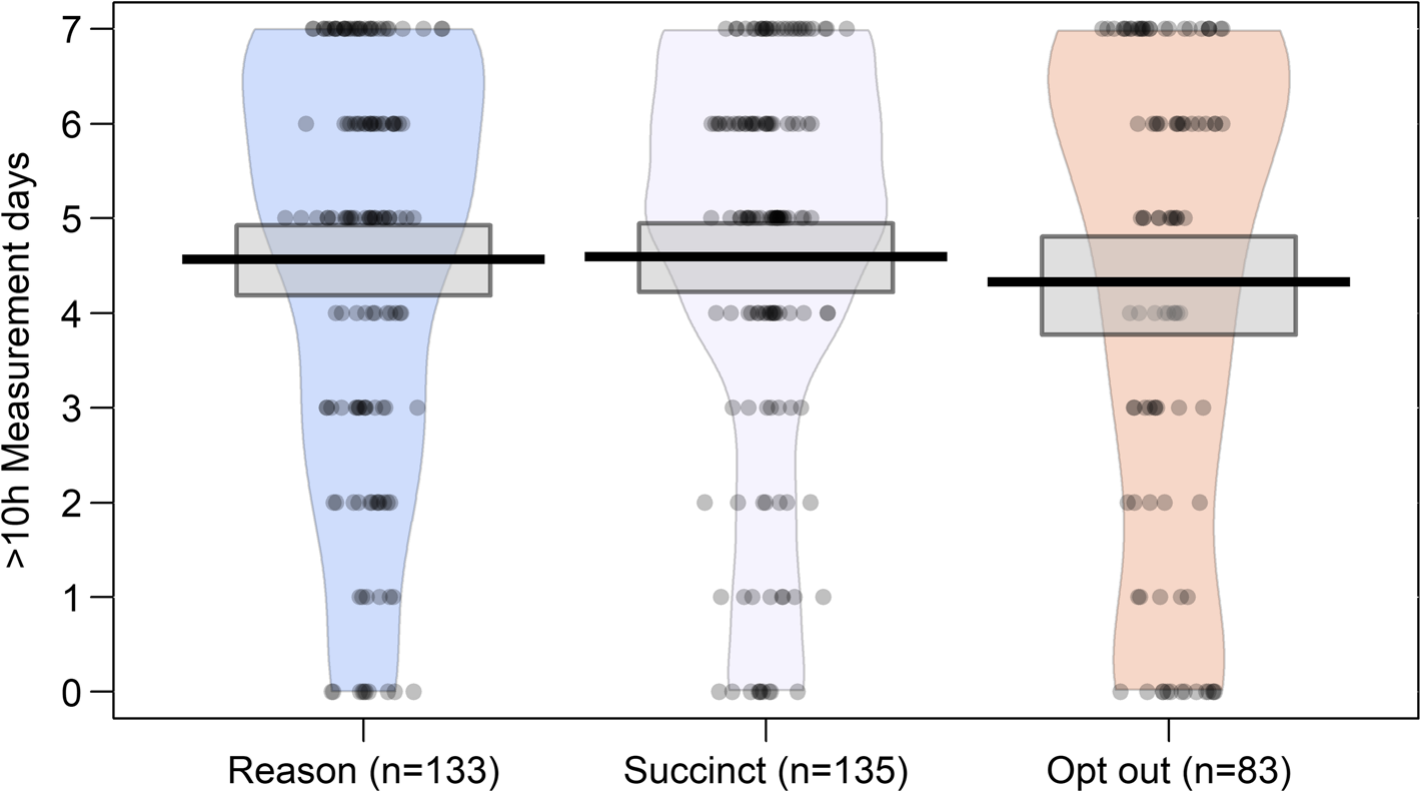
Measurement days of > 10 h of data gathered by group. Horizontal lines represent means, boxes Bayesian 95% Highest Density Intervals (with flat priors)

Figure 7 shows densities and spread of valid measurement days by group. As can be visually inspected from the HDIs, population means are equivalent.

Differences between the distributions of measurement days with > 10 h of data were not detected between the reason and succinct groups, χ^2^(7) = 7.893, *p* = 0.342. A Bayesian contingency tables test provided BF_01_ = 6.96 (Poisson sampling, prior concentration = 1.0; prior robustness test depicts a concave function where, as concentration approaches 2, BF_01_ approaches 22.97).

Differences were not detected in valid wear day distributions between participants for whom reminders were sent, and for whom they were not: χ^2^(7) = 8.344, *p* = 0.303. A BF_01_ = 34.79 (Poisson sampling, prior concentration = 1.0; robustness function is concave as before. As concentration approaches 2, BF_01_ approaches 93.50).

Again, equivalence tests of mean differences between message types (− 0.07 days, 90% CI [− 0.47; 0.33]) was statistically significantly larger than d = − 0.3 and smaller than d = 0.3. The mean difference between reminder and opt out groups (− 0.18 days, 90% CI [− 0.60; 0.24]) was statistically significantly smaller than d = 0.3, but we could not reject the hypothesis that the effect was higher than d = − 0.3.

A MANOVA with both total wear time minutes and valid wear days as dependent variables neither detected differences between the reason, succinct and opt out groups (*F*(4, 682) = 2.335, *p* = 0.054, Wilk’s Λ = 0.973), although multicollinearity may have posed a problem to the model (τ = 0.81, ρ = 0.93).

#### Dose dependence

**Fig. 8:**
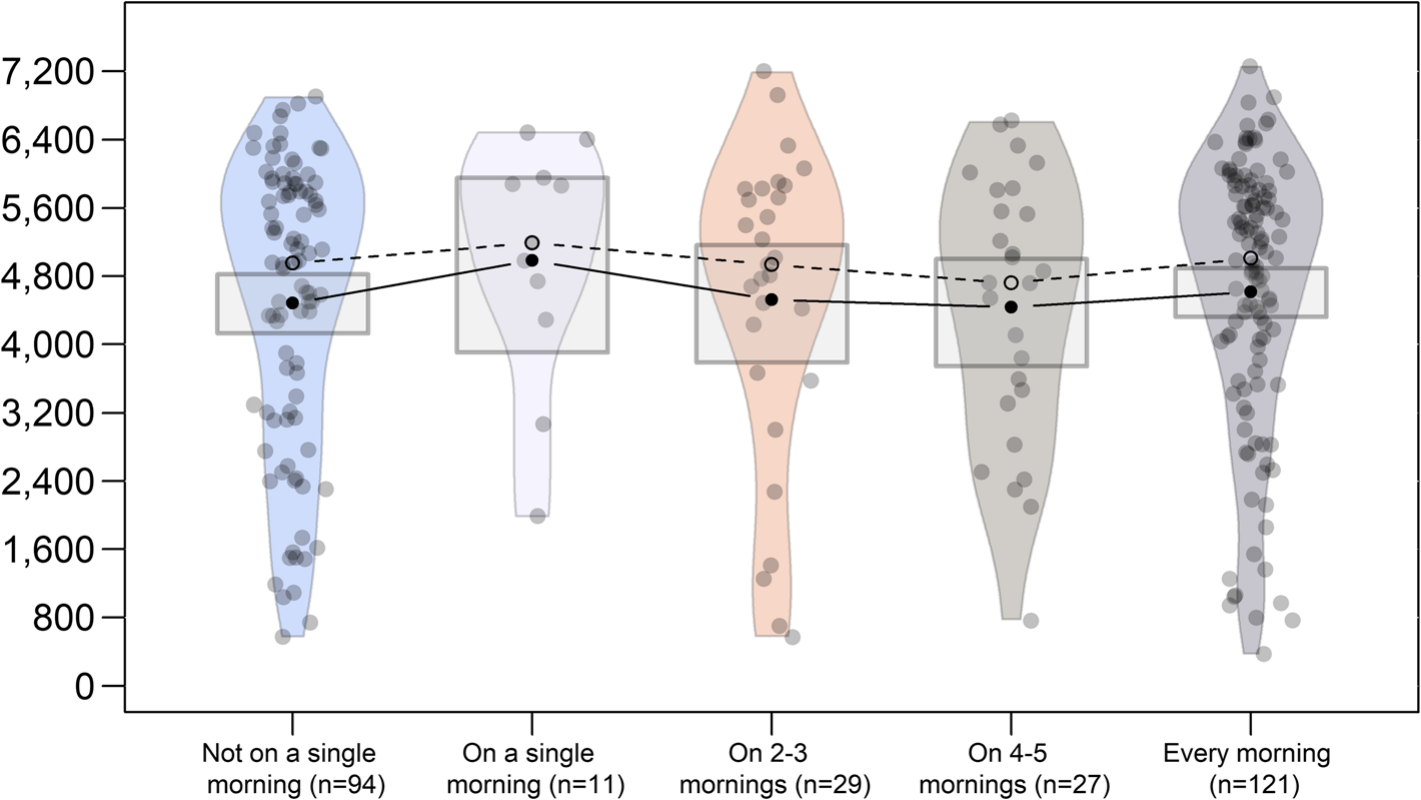
Self-reported opening and reading of messages. Y-axis is total wear time. Boxes represent 95% HDIs for the means, solid lines connect means and dashed lines connect medians. Participants who opted out of reminders are aggregated with those who indicated not having opened the messages even once. Participants who received messages, but did not answer the question on message reading, are excluded

If reading of messages is linearly related to wear time, an upward moving slope in means would have been expected. The dose dependence curve Fig. 8 is flat, showing no support for such a relationship between messages and wear time.

## Discussion

In an attempt to improve measurement of physical activity and sedentary behaviour—key public health issues—this study evaluated the effects of two interventions to increase accelerometer wear times during the first two recruitment waves of the Let’s Move It trial. Specifically, it tested the effects of the because-heuristic on accelerometer wear time in older adolescents. We did not detect increased wear times among participants who received a reason in their daily SMS reminders, nor did we detect different wear times between those receiving the reminder messages and those who opted out. In all cases, null models were supported over those with small-to-medium sized effects (see supplementary website (https://git.io/vNl8X) sections “Interpreting Bayes Factors” and “Bayesian ANOVA” for details). As it is neither logically nor statistically appropriate to conclude the absence of an effect from a non-significant hypothesis test [60, 61], we hope the analyses contribute to a long-overdue inferential development in the field.

Our main results are in line with the results of Belton et al. [11], of reminders not being able to increase wear time, despite attempting to improve on the earlier studies by not having exactly the same message sent every day. We do not have data on whether the reminder caused our participants put on the accelerometer more often, in spite of not increasing wear time as found in [11].

Although the xerox machine study [12] has been highly publicised for 30 years, the contextual framework of the effect remains unclear. Thus, many possible reasons could explain the null results obtained in this study, including the impersonal nature of SMS communication (as compared to face-to-face interaction), the source of the information, being incapable to complete the requested task, and a several other factors varying in plausibility – demographic factors, the target behaviour, contextualised cognitive processes and so forth. Accordingly, the effect of reasons on this particular behavior, given our context and delivery method, has proved smaller than what would be considered minimally interesting (although participants did attribute remembering to wear their accelerometer to receipt of the reminders), and possibly zero. Thus, the *naïve because-heuristic* does not receive support in the current study. We can not make conclusions regarding the weaker claim of reasons affecting only tasks which are easy to carry out, due to design and sample size considerations.

The flat dose-dependence curve can indicate several things, including the possibility that text messages do not affect wear times. Attributing remembering to getting reminded could be a case of a post hoc reasoning error [62]. Another possibility is that the messages could have had a small effect, but opening and reading the message provided no additional benefit. For example, the participant could have looked at the preview of the message on the cell phone screen and remembered without reading the whole message.

As there were no differences between the SMS and no-SMS arms, this effect may have been masked by selection bias, with those people who expect to experience problems with remembering, opting in to receive SMS reminders. As consent was almost fully dependent on the recruitment prompt, an additional assumption is needed that the two recruitment waves differ qualitatively (on an unobserved confounder). So, for example, the second wave may have consisted of more compliant participants or the potential interactions with the first wave participants might have made the opinion of the study more favorable. Thirdly, the effect of reminders may not have been linear, or only a small dose is needed to form a habit, and thus achieve maximal effect. This explanation requires the same assumptions as the one described above. Fourth, the flat curve may also be caused by unreliable measurement: dose should be operationalized in a way not dependent on self-report. Finally, it is possible that receiving reminders causes an undermining of one’s own responsibility, so that those who receive reminders relinquish control and do not carry out the remembering techniques (e.g. placing of the accelerometer in a conspicuous place as a prompt to put it on) they would have, in the absence of reminders.

It may be that daily accelerometer wear is not determined by heuristic/automatic processes, but rather, is under more reflective reasoning processes. In this case, these reminders should have provided justifications and rationales that truly are important for this target group. We do not have any evidence what thoughts and connotations our reminder content evoked in the youth’s minds, and whether it was counterproductive. Finally, it is possible that participants who had agreed to take part in the accelerometer data collection already had made the reflective decision and proceeded to “implemental mindset” where persuasion messages are less relevant; e.g. as speculated in [63].

### Limitations and strengths

There are a number of ways this study could have been improved on.

#### Opening and reading the messages (manipulation success)

Number of participants who opened and read the messages was assessed with a questionnaire instead of objective log data. This self-report measure (as well as the other post-intervention questionnaire items) was only a non-validated single item, thus probably far from optimal in terms of reliability. We had no reliable way to certify at which times the messages were received or whether they were opened at all. Anecdotal evidence indicated that the messages were too late for some students (i.e. they had already left the house and forgotten the accelerometer when receiving the message). On the other hand, we deemed sending the messages too early might pose an acceptability issue. The SMS queue in the gateway device presented a difficulty: larger number of message recipients heavily affected the deviation of delivery times, making the last messages in the queue arrive late for some students. During the second recruitment wave, time of initiating the send process was changed to be 45 min earlier (06:15 instead of 07:00), but we do not have data on the effect of this change.

We attempted to alleviate effects of not opening the messages by starting the each with the word “because”, so that message preview would render it visible on many devices even when not opened. Unfortunately we did not have access to a gateway system that could have sent e.g. MMS-messages, where a small picture could have been added, thus providing log data on how many times the picture was downloaded.

#### Contamination effects and masking the different message conditions

Participants may have found out their group allocation when discussing the messages with peers. This would require the discussion to have been about the nuances of message content and assumes that the participants are intrigued enough to spend time on making such inferences in the first place; an assumption perhaps not warranted. It is unclear how the discovery of SMS arm would have affected the results, but the possibility of confounding cannot be excluded. Randomising the groups by clusters could have helped to avoid this, but would have led to a reduction in statistical power. Still, the participants reported mainly not having discussed the messages with peers.

#### Sampling plan

The stopping rule for data collection was not defined in advance. The decision to collect another wave of participants with the same design was made, when it became apparent that the messages did not have the strong impact we had anticipated. This leads to uninformative *p*-values in terms of error control [64], whereas Bayesian analyses are not as crucially affected by stopping rules ([65], but see also [66]).

#### Lack of a randomised no-SMS control group

In order to avoid distortion of main trial outcomes (e.g. increased PA), care had to be taken in this within-trial RCT. The risk of sabotage due to disappointment of being allocated to a no-SMS control group was deemed too high, and thus participants were not randomised into a no-SMS group. This, in turn, lessens the strength of conclusions based on wear times between the participants receiving the reminder and those not receiving one. People who know they do not need a reminder may have thus ended up self-selecting to the no-SMS group.

This presumes that teenagers studying in a vocational school have the capacity to make accurate predictions about their future self-regulation capabilities in an unfamiliar task (putting on an accelerometer). On the other hand, as described, the wording of the recruitment prompt was slightly modified from wave 1 to wave 2, and consent to reminders was increased from 53% (85 out of 97) to 95% (176 out of 186), whereas wear times did not differ. Thus, strong selection effects seem unlikely. Although this indicates that opting out was more a result of the recruitment procedure than knowledge of not needing the reminders, future research should aim to randomise when feasible.

One way to address this problem would have been an n-of-1 design, where each day is randomised to one of the three message conditions. With this design, one should be careful to not leave learning effects undetected, as participants could habituate to reminders and forget in the concurrent absence of them.

#### Message content and size of request

The intervention was not piloted, nor was extensive testing of it’s component parts done, which may have affected the results. The pre-testing of the message content was limited, too, and we thus do not have data on whether our participants considered the messages persuasive. This could be important theoretically, especially *if* the request size was considered large *and* our reasons were perceived as placebic or near-placebic. However, this might not be an issue in the first place, as participants had already agreed to wear the accelerometer as part of the trial. Message content (as explicated in hypothesis H_2_) may not play a role at all, if the real reason for non-compliance is e.g. leaving the house in a rush. In such a case, though, we would still expect those who are reminded to have increased wear times compared to those who are not reminded (hypothesis H_1_).

### Pre-registration

In this paper, we attempted to answer to the call of more stringent methodology by pre-registration. Optimally, this would have been done prior to beginning data collection. In these cases, it has been proposed that analyses should be considered exploratory [67]—especially in the presence of researcher degrees of freedom or data-dependent analysis decisions [68]—and can render *p*-values meaningless. In our case, this mistake turned out to be nonconsequential. We used Bayes factors to avoid claiming findings based on *p*-values alone, as recently warned against by the American Statistical Association [69]. Other approaches we used to address the replicability problem were transparent reporting and open data.

### Implications for practice

Our results, in line with some other studies e.g. [11] indicate that researchers should not expect simple reminders to have strong effects on accelerometer wear times among youth. Also, despite previous strong claims, the because-heuristic in this context lacks the strength attributed to it in the popular literature. When considering using SMS reminders for youth, we suggest ensuring that remembering plausibly plays the key role in compliance with the behavior and target group in question, instead of other determinants/factors (such as social norms or motivation). Participants’ coping skills and attention span may act as a ceiling to the potential effect of the reminder in situations where the target behavior can not immediately be carried out, so suitability of SMS reminders could be assessed in these respects as well.

### Implications for future research

To an extent, the findings here apply to situations where cost-effective reminders can potentially improve compliance. These areas may range from medication adherence [70] to sunscreen use [71]. An interesting hypothesis to test, would be whether reminders actually *reduce* active coping strategies that people use spontaneously – this could partly explain some null findings in the literature on technical reminder systems [72]. Second, the delivery of the reminders should optimally be objectively trackable, in order to make firm conclusions about the independent effects of delivery and receipt. Third, the context (including timing and location) where the participant receives the reminder is likely to be important, as well as the coping behaviour of the control group. It may also be worthwhile to gauge whether altering frequency of reminders affects the target behavior [70], or if the system can be made such that it adapts to the users and their environments [73]. Lastly, it might be worthwhile to investigate, if personally meaningful persuasive arguments work better than vague and general ones (e.g. contributing to science), which were used in order to minimise risk of participants changing their activity behaviour instead of merely the wear time behaviour. As the literature presented earlier suggests, *any* reasons should be enough for heuristic decision making, whereas good reasons may be needed for more reflective decisions. Further theorising and additional measures to test hypotheses based on dual process models may be fruitful – but we encourage researchers to see [74–79], and also consider a wider perspective from complexity and systems theory [80–83], which have recently been applied in public health [84–86].

## Conclusion

In this research, we have found evidence against the assumed superiority of the naïve because-heuristic; providing reasons in simple compliance requests having a general persuasive effect on behaviour. By using Bayesian methods and equivalence testing, we were able to claim evidence of no effect for the because heuristic in this setting. Likewise, sending SMS reminders was not associated with improved accelerometer wear times. Although we did not randomise the no-SMS group, the changed recruitment procedure plausibly accounts for majority of the selection effect, and a more potent explanation for the lack of differing weartimes is reaching the ceiling of the participants’ ability to wear in the absence of very high motivation.

Our design had several limitations, which should be improved upon in future research. All in all, we remain pessimistic of the efficacy of the naïve because-heuristic and of simple reminders, even if they have a potent effect in participants’ perceptions.

We conclude that despite strong claims, there is reason to consider the study of the because-heuristic a degenerating research programme [87], although there may be some contexts where the technique works as intended. Seeking to increase accelerometry wear time in participants may benefit from a design using the intervention mapping approach [88], including a plausible theoretical framework.

## Additional files

Additional file 1: **Appendix S1.** Post-SMS questionnaire (Finnish). (DOCX 16 kb)

Additional file 2: **Appendix S2.** Post-SMS questionnaire (English translation) (DOCX 31 kb)

Additional file 3: **Appendix S3.** CRediT – contributor role taxonomy (DOCX 63 kb)

## Abbreviations

BF: Bayes Factor
LMI: Let’s Move It intervention to increase physical activity and decrease sedentary behavior in older adolescents
RCT: Randomised controlled trial
SMS: Short Message Service, also known as “text messaging”

## References

[1] Cain KL, Sallis JF, Conway TL, Van Dyck D, Calhoon L (2013). Using accelerometers in youth physical activity studies: a review of methods. J Phys Act Health.

[2] Matthews CE, Hagströmer M, Pober DM, Bowles HR (2012). Best practices for using physical activity monitors in population-based research. Med Sci Sports Exerc.

[3] Prince SA, Adamo KB, Hamel ME, Hardt J, Gorber SC, Tremblay M (2008). A comparison of direct versus self-report measures for assessing physical activity in adults: a systematic review. Int J Behav Nutr Phys Act.

[4] Zhuang J, Chen P, Wang C, Huang L, Zhu Z, Zhang W (2013). Characteristics of missing physical activity data in children and youth. Res Q Exerc Sport.

[5] Ward DS, Evenson KR, Vaughn A, Rodgers AB, Troiano RP (2005). Accelerometer use in physical activity: best practices and research recommendations. Med Sci Sports Exerc.

[6] Audrey S, Bell S, Hughes R, Campbell R. Adolescent perspectives on wearing accelerometers to measure physical activity in population-based trials. Eur J Pub Health. 2012;cks081:475-80. 10.1093/eurpub/cks081 23132872

[7] Sirard JR, Slater ME (2009). Compliance with wearing physical activity accelerometers in high school students. J Phys Act Health.

[8] Sallis JF, Saelens BE, Frank LD, Conway TL, Slymen DJ, Cain KL (2009). Neighborhood built environment and income: examining multiple health outcomes. Soc Sci Med.

[9] Barak S, Wu SS, Dai Y, Duncan PW, Behrman AL (2014). Adherence to Accelerometry measurement of community ambulation poststroke. Phys Ther.

[10] Toftager M, Kristensen PL, Oliver M, Duncan S, Christiansen LB, Boyle E (2013). Accelerometer data reduction in adolescents: effects on sample retention and bias. Int J Behav Nutr Phys Act.

[11] Belton S, O’Brien W, Wickel EE, Issartel J (2013). Patterns of non-compliance in adolescent field based accelerometer research. J Phys Act Health.

[12] Langer EJ, Blank A, Chanowitz B (1978). The mindlessness of ostensibly thoughtful action: the role of “placebic” information in interpersonal interaction. J Pers Soc Psychol.

[13] Pratkanis AR, Pratkanis AR (2007). Social influence analysis: an index of tactics. The science of social influence: advances and future progress.

[14] Cialdini RB, Goldstein NJ, Martin SJ (2009). Influence: Science and practice.

[15] Blount J. Fanatical prospecting: the ultimate guide to opening sales conversations and filling the pipeline by leveraging social selling, telephone, email, text, and cold calling. New Jersey: John Wiley & Sons; 2015.

[16] Goldman B (2008). The Science of Settlement: Ideas for Negotiators.

[17] Mortensen KW (2013). Maximum influence: the 12 universal Laws of power persuasion.

[18] Weinschenk S. The power of the word “because” to get people to do stuff. Psychol Today. 2013; https://www.psychologytoday.com/blog/brain-wise/201310/the-power-the-word-because-get-people-do-stuff. Accessed 5 Nov 2015.

[19] Cialdini RB (2001). Influence: Science and practice. 4th edition.

[20] Key SM, Edlund JE, Sagarin BJ, Bizer GY (2009). Individual differences in susceptibility to mindlessness. Personal Individ Differ.

[21] Folkes VS (1985). Mindlessness or mindfulness: a partial replication and extension of Langer, blank, and Chanowitz. J Pers Soc Psychol.

[22] Langer EJ, Chanowitz B, Blank A (1985). Mindlessness–mindfulness in perspective: a reply to Valerie Folkes. J Pers Soc Psychol.

[23] Makel MC, Plucker JA, Hegarty B. Replications in psychology research how often do they really occur? Perspect Psychol Sci. 2012;7:537–42. 10.1177/1745691612460688 26168110

[24] Pollock CL, Smith SD, Knowles ES, Bruce HJ (1998). Mindfullness limits compliance with the That’s-not-all technique. Personal Soc Psychol Bull.

[25] Slugoski BR (1995). Mindless processing of requests? Don’t ask twice. Br J Soc Psychol.

[26] Laaksonen M, Talala K, Martelin T, Rahkonen O, Roos E, Helakorpi S (2008). Health behaviours as explanations for educational level differences in cardiovascular and all-cause mortality: a follow-up of 60 000 men and women over 23 years. Eur J Pub Health.

[27] Elgar FJ, Pförtner T-K, Moor I, De Clercq B, Stevens GWJM, Currie C (2015). Socioeconomic inequalities in adolescent health 2002–2010: a time-series analysis of 34 countries participating in the health behaviour in school-aged children study. Lancet.

[28] National institute for Health and Welfare. School health survey 2015 results: Lifestyle. Terveyden ja hyvinvoinnin laitos. 2015. https://web.archive.org/web/20170306230805/https://www.thl.fi/fi/tutkimus-ja-asiantuntijatyo/vaestotutkimukset/kouluterveyskysely/tulokset/tulokset-aiheittain/elintavat. Accessed 4 Dec 2015.

[29] Hankonen N, Heino MTJ, Araujo-Soares V, Sniehotta FF, Sund R, Vasankari T (2016). ‘Let’s move it’ – a school-based multilevel intervention to increase physical activity and reduce sedentary behaviour among older adolescents in vocational secondary schools: a study protocol for a cluster-randomised trial. BMC Public Health.

[30] Hankonen N, Heino MTJ, Hynynen S-T, Laine H, Araújo-Soares V, Sniehotta FF, et al. Randomised controlled feasibility study of a school-based multi-level intervention to increase physical activity and decrease sedentary behaviour among vocational school students. Int J Behav Nutr Phys Act. 2017;14. 10.1186/s12966-017-0484-0. 10.1186/s12966-017-0484-0 PMC5361824 28327174

[31] Heino MTJ. No use reasoning with adolescents? A randomised controlled trial comparing persuasive messages. 2016. https://helda.helsinki.fi/handle/10138/163800. Accessed 7 Jun 2017.

[32] Heino MTJ. Comparing persuasive SMS reminders: supplementary website. 2018. https://web.archive.org/web/20180828180801/https://heinonmatti.github.io/sms-persuasion/sms-persuasion-supplement.html. Accessed 21 Feb 2018.

[33] Rowlands, A. V., Fraysse, F., Catt, M., Stiles, V. H., Stanley, R. M., Eston, R. G., & Olds, T. S. Comparability of Measured Acceleration from Accelerometry-Based Activity Monitors. Medicine & Science in Sports & Exercise, 2015;47(1), 201–210. 10.1249/MSS.0000000000000394 10.1249/MSS.0000000000000394 24870577

[34] Aittasalo M, Vähä-Ypyä H, Vasankari T, Husu P, Jussila A-M, Sievänen H (2015). Mean amplitude deviation calculated from raw acceleration data: a novel method for classifying the intensity of adolescents’ physical activity irrespective of accelerometer brand. BMC Sports Sci Med Rehabil.

[35] Vähä-Ypyä H, Vasankari T, Husu P, Suni J, Sievänen H (2015). A universal, accurate intensity-based classification of different physical activities using raw data of accelerometer. Clin Physiol Funct Imaging.

[36] Dienes Z. Understanding psychology as a science: an introduction to scientific and statistical inference. New York: Palgrave Macmillan; 2008.

[37] Wagenmakers E-J (2007). A practical solution to the pervasive problems of p values. Psychon Bull Rev.

[38] Fanelli D (2013). Only reporting guidelines can save (soft) science. Eur J Personal.

[39] Boutron I, Moher D, Altman DG, Schulz KF, Ravaud P (2008). Extending the CONSORT statement to randomized trials of nonpharmacologic treatment: explanation and elaboration. Ann Intern Med.

[40] Allen L, Scott J, Brand A, Hlava M, Altman M (2014). Publishing: credit where credit is due. Nature.

[41] R Core Team (2015). R: a language and environment for statistical computing.

[42] RStudio Team (2015). RStudio: Integrated Development Environment for R.

[43] Wickham H (2009). ggplot2: elegant graphics for data analysis.

[44] Phillips N. yarrr: A companion to the e-book YaRrr!: The Pirate’s Guide to R. 2016. https://cran.r-project.org/web/packages/yarrr/vignettes/pirateplot.html.

[45] Baguley T (2012). Serious stats: a guide to advanced statistics for the behavioral sciences.

[46] Bowman AW, Azzalini A. R package sm: nonparametric smoothing methods (version 2.2–5.4). University of Glasgow, UK and Università di Padova, Italia; 2014. URL https://cran.r-project.org/web/packages/sm/sm.pdfhttp://azzalini.stat.unipd.it/Book_sm.

[47] Peters G.-J. Y. userfriendlyscience: Quantitative analysis made accessible. 2016. http://CRAN.R-project.org/package=userfriendlyscience.

[48] Morey RD, Hoekstra R, Rouder JN, Lee MD, Wagenmakers E-J. The fallacy of placing confidence in confidence intervals. Psychon Bull Rev. 2015. 10.3758/s13423-015-0947-8. 10.3758/s13423-015-0947-8 PMC4742505 26450628

[49] Heino MTJ, Vuorre M, Hankonen N. Bayesian evaluation of behavior change interventions: A brief introduction and a practical example. PsyArXiv. 2017. doi:10.17605/OSF.IO/XMGWV. 10.1080/21642850.2018.1428102 PMC8114380 34040821

[50] Morey RD, Romeijn J-W, Rouder JN. The philosophy of Bayes factors and the quantification of statistical evidence. J Math Psychol. 2016. 10.1016/j.jmp.2015.11.001.

[51] Etz A, Vandekerckhove J. Introduction to Bayesian Inference for Psychology. Psychon Bull Rev. 2018;25:5–34. 10.3758/s13423-017-1262-3 28378250

[52] Etz A, Vandekerckhove J (2016). A Bayesian perspective on the reproducibility project: psychology. PLoS One.

[53] Morey RD, Rouder JN. BayesFactor: Computation of Bayes Factors for Common Designs. 2015. https://CRAN.R-project.org/package=BayesFactor.

[54] Richard FD, Bond CF, Stokes-Zoota JJ (2003). One hundred years of social psychology quantitatively described. Rev Gen Psychol.

[55] Jamil T, Ly A, Morey RD, Love J, Marsman M, Wagenmakers E-J. Default “Gunel and dickey” Bayes factors for contingency tables. Behav Res Methods. 2015:1–15. 10.3758/s13428-016-0739-8 PMC5405059 27325166

[56] Lakens D (2017). Equivalence tests: a practical primer for t tests, correlations, and meta-analyses. Soc Psychol Personal Sci.

[57] Lakens D (2016). TOSTER: two one-sided tests (TOST) equivalence testing.

[58] Gelman A, Carlin J (2014). Beyond power calculations assessing type S (sign) and type M (magnitude) errors. Perspect Psychol Sci.

[59] Davis-Stober CP, Dana J (2013). Comparing the accuracy of experimental estimates to guessing: a new perspective on replication and the “crisis of confidence” in psychology. Behav Res Methods.

[60] Lakens D, McLatchie N, Isager PM, Scheel AM, Dienes Z. Improving Inferences about Null Effects with Bayes Factors and Equivalence Tests. J Gerontol B. Psychol Sci Soc Sci. 2018;:gby065. 10.1093/geronb/gby065 29878211

[61] Harms C, Lakens D. Making “Null Effects” Informative: Statistical Techniques and Inferential Frameworks. J Clin Transl Res in press. doi:10.31234/osf.io/48zca. PMC6412612 30873486

[62] Hansen H. Fallacies. In: Zalta EN, editor. The Stanford encyclopedia of philosophy. Summer 2015. 2015. https://plato.stanford.edu/entries/fallacies/. Accessed 12 Mar 2016.

[63] Armor DA, Taylor SE (2003). The effects of mindset on behavior: self-regulation in deliberative and implemental frames of mind. Personal Soc Psychol Bull.

[64] Sagarin BJ, Ambler JK, Lee EM (2014). An ethical approach to peeking at data. Perspect Psychol Sci.

[65] Dienes Z (2014). Using Bayes to get the most out of non-significant results. Quant Psychol Meas.

[66] Simonsohn U (2014). Posterior-Hacking: Selective reporting invalidates Bayesian results also. SSRN scholarly paper.

[67] Wagenmakers E-J, Wetzels R, Borsboom D, van der Maas HLJ, Kievit RA (2012). An agenda for purely confirmatory research. Perspect Psychol Sci.

[68] Gelman A, Loken E (2014). The statistical crisis in science. Am Sci.

[69] Wasserstein RL, Lazar NA. The ASA’s Statement on p-Values: Context, Process, and Purpose. The American Statistician. 2016;70:129–33.

[70] Pop-Eleches C, Thirumurthy H, Habyarimana JP, Zivin JG, Goldstein MP, Walque DD (2011). Mobile phone technologies improve adherence to antiretroviral treatment in a resource-limited setting: a randomized controlled trial of text message reminders. AIDS Lond Engl.

[71] Armstrong AW, Watson AJ, Makredes M, Frangos JE, Kimball AB, Kvedar JC (2009). Text-message reminders to improve sunscreen use: a randomized, controlled trial using electronic monitoring. Arch Dermatol.

[72] Demonceau J, Ruppar T, Kristanto P, Hughes DA, Fargher E, Kardas P (2013). Identification and assessment of adherence-enhancing interventions in studies assessing medication adherence through electronically compiled drug dosing histories: a systematic literature review and meta-analysis. Drugs.

[73] Hekler EB, Klasnja P, Riley WT, Buman MP, Huberty J, Rivera DE (2016). Agile science: creating useful products for behavior change in the real world. Transl Behav Med.

[74] Evans JSBT, Stanovich KE (2013). Dual-process theories of higher cognition: advancing the debate. Perspect Psychol Sci.

[75] Keren G (2013). A tale of two systems: a scientific advance or a theoretical stone soup? Commentary on Evans & Stanovich (2013). Perspect Psychol Sci.

[76] Kruglanski AW (2013). Only one? The default interventionist perspective as a Unimodel—commentary on Evans & Stanovich (2013), only one? The default interventionist perspective as a Unimodel—commentary on Evans & Stanovich (2013). Perspect Psychol Sci.

[77] Bellini-Leite SC (2018). Dual process theory: systems, types, minds, modes, kinds or metaphors? A critical review. Rev Philos Psychol.

[78] Mugg J (2016). The dual-process turn: how recent defenses of dual-process theories of reasoning fail. Philos Psychol.

[79] Westfall J, Yarkoni T (2016). Statistically controlling for confounding constructs is harder than you think. PLoS One.

[80] Rickles D, Hawe P, Shiell A (2007). A simple guide to chaos and complexity. J Epidemiol Community Health.

[81] Diez Roux AV (2011). Complex systems thinking and current impasses in health disparities research. Am J Public Health.

[82] Hawe P, Shiell A, Riley T (2009). Theorising interventions as events in systems. Am J Community Psychol.

[83] Hawe P (2015). Lessons from complex interventions to improve health. Annu Rev Public Health.

[84] Carey G, Malbon E, Carey N, Joyce A, Crammond B, Carey A (2015). Systems science and systems thinking for public health: a systematic review of the field. BMJ Open.

[85] Rutter H, Savona N, Glonti K, Bibby J, Cummins S, Finegood DT (2017). The need for a complex systems model of evidence for public health. Lancet.

[86] Sniehotta FF, Araújo-Soares V, Brown J, Kelly MP, Michie S, West R (2017). Complex systems and individual-level approaches to population health: a false dichotomy?. Lancet Public Health.

[87] Lakatos I. History of science and its rational reconstructions: Springer; 1971. http://link.springer.com/chapter/10.1007/978-94-010-3142-4_7. Accessed 2 Dec 2015

[88] Bartholomew Eldredge LK, Markham CM, Ruiter RA, Fernández ME, Kok G, Parcel GS. Planning health promotion programs: an intervention mapping approach. New Jersey: John Wiley & Sons; 2016.

