# Supplementary figures and images for: Simple and rationale-providing SMS reminders to promote accelerometer use: a within-trial randomised trial comparing persuasive messages
Source: BMC Public Health. 2018 Dec 7;18:1352. doi: 10.1186/s12889-018-6121-2 (PMC6286544; doi:10.1186/s12889-018-6121-2)

Appendix S1: Post-SMS questionnaire (Finnish).


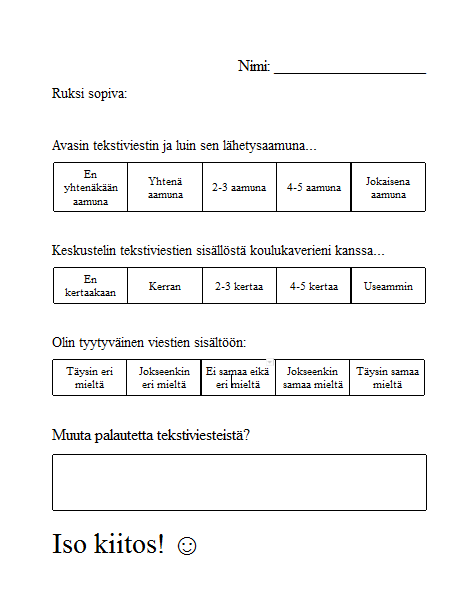

Supplement: Supplementary file 1 — Appendix S1. Post-SMS questionnaire (Finnish). (DOCX 16 kb) [file 12889_2018_6121_MOESM1_ESM.docx]

Appendix S2: Post-SMS questionnaire (English translation)


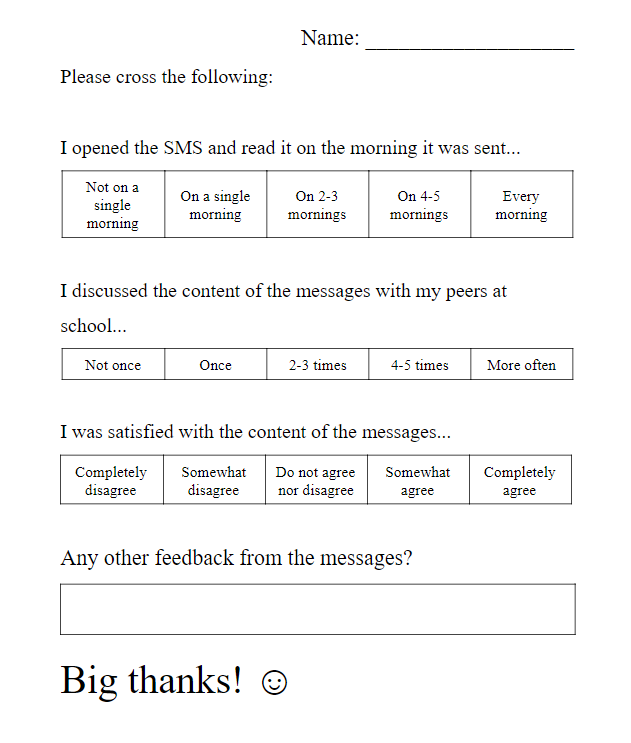

Supplement: Supplementary file 2 — Appendix S2. Post-SMS questionnaire (English translation) (DOCX 31 kb) [file 12889_2018_6121_MOESM2_ESM.docx]
