# Supplementary material for: Simple and rationale-providing SMS reminders to promote accelerometer use: a within-trial randomised trial comparing persuasive messages
Source: BMC Public Health. 2018 Dec 7;18:1352. doi: 10.1186/s12889-018-6121-2 (PMC6286544; doi:10.1186/s12889-018-6121-2)
Supplement: Supplementary file 3 — Appendix S3. CRediT – contributor role taxonomy (DOCX 63 kb) [file 12889_2018_6121_MOESM3_ESM.docx]

Appendix S3: CRediT – contributor role taxonomy

| **Taxonomy category** | **Description** | **Author responsible** |
| --- | --- | --- |
| Study conception | Ideas; formulation of research question; statement of hypothesis. | Nelli Hankonen & Matti Heino |
| Methodology | Development or design of methodology; creation of models. | Matti Heino, Nelli Hankonen, Keegan Knittle, Ari Haukkala |
| Computation | Programming, software development; designing computer programs; implementation of the computer code and supporting algorithms. | UKK-institute, Kryptoniitti joint-stock company |
| Formal analysis | Application of statistical, mathematical or other formal techniques to analyse study data. | Matti Heino |
| Investigation: performed the experiments | Conducting the research and investigation process, specifically performing the experiments. | Matti Heino & Let’s Move It data collection team |
| Investigation: data/evidence collection | Conducting the research and investigation process, specifically data/evidence collection. | Let’s Move It data collection team |
| Resources | Provision of study materials, reagents, materials, patients, laboratory samples, animals, instrumentation or other analysis tools. | Tommi Vasankari, Nelli Hankonen |
| Data curation | Management activities to annotate (produce metadata) and maintain research data for initial use and later re-use. | Matti Heino |
| Writing/manuscript preparation: writing the initial draft | Preparation, creation and/or presentation of the published work, specifically writing the initial draft. | Matti Heino |
| Writing/manuscript preparation: critical review, commentary or revision | Preparation, creation and/or presentation of the published work, specifically critical review, commentary or revision. | Matti Heino, Nelli Hankonen, Ari Haukkala, Keegan Knittle, Tommi Vasankari |
| Writing/manuscript preparation: visualization/data presentation | Preparation, creation and/or presentation of the published work, specifically visualization/data presentation. | Matti Heino |
| Supervision | Responsibility for supervising research; project orchestration; principal investigator or other lead stakeholder. | Nelli Hankonen |
| Project administration | Coordination or management of research activities leading to this publication. | Nelli Hankonen |
| Funding acquisition | Acquisition of the financial support for the project leading to this publication. | Nelli Hankonen, Ari Haukkala, Tommi Vasankari, the UKK-institute |

[40]

40. Allen L, Scott J, Brand A, Hlava M, Altman M. Publishing: Credit where credit is due. Nature. 2014;508:312–3.
